# Genomic Signatures in Maned Three‐Toed Sloths From Ancient to Recent Environmental Changes in Brazil's Threatened Atlantic Forest

**DOI:** 10.1111/mec.70148

**Published:** 2025-10-18

**Authors:** Larissa S. Arantes, Diego De Panis, Flávia R. Miranda, Fabrício R. Santos, Michael Hiller, Camila J. Mazzoni

**Affiliations:** ^1^ Department of Evolutionary Genetics Leibniz Institute for Zoo and Wildlife Research (IZW) Berlin Germany; ^2^ Berlin Center for Genomics in Biodiversity Research (BeGenDiv) Berlin Germany; ^3^ Department of Biological Sciences State University of Santa Cruz (UESC) Ilhéus Brazil; ^4^ Instituto de Pesquisa e Conservação de Tamanduás no Brasil (IPCTB) Campo Grande Brazil; ^5^ Department of Genetics, Ecology and Evolution Federal University of Minas Gerais (UFMG) Belo Horizonte Brazil; ^6^ LOEWE Centre for Translational Biodiversity Genomics Frankfurt Germany; ^7^ Senckenberg Research Institute Frankfurt Germany; ^8^ Institute of Cell Biology and Neuroscience, Faculty of Biosciences Goethe University Frankfurt Germany

**Keywords:** *Bradypus crinitus*, *Bradypus torquatus*, deforestation, demographic analysis, genomics, inbreeding

## Abstract

Environmental and climatic changes have shaped the evolutionary trajectories of natural populations, leaving genomic signatures that reflect how species respond to these shifts and their impacts on genetic health. While these insights are essential for unravelling evolutionary histories and informing conservation strategies, studies on Neotropical species remain largely underrepresented. Maned Three‐Toed Sloths, endemic to the fragmented Atlantic Forest of Brazil, were recently reclassified into two distinct species: the Northern and Southern Maned Sloths. Our study investigates the genomic imprints left by ancient and recent environmental changes in the Atlantic Forest in these two sloth lineages, using whole‐genome resequencing data. Our analysis reveals that the Southern Maned Sloth exhibits a smaller historical population size than the Northern Maned Sloth. This disparity likely stems from differing climatic changes along the Atlantic Forest distribution during the Pleistocene, characterised by greater climatic stability and larger refugia areas in the north. Consequently, the southern lineage presents a lower genetic diversity and higher overall inbreeding level. Nonetheless, the northern population has experienced a fast increase in inbreeding levels in the last few decades, likely associated with extensive recent deforestation in the northeast region of Bahia State. The distinct demographic trajectories also resulted in the northern lineage carrying a higher genetic load, implying higher fitness costs for this lineage if inbreeding persists. Together, our findings confirm the independent evolutionary paths of these two lineages and underscore the unique conservation challenges posed by both historical climatic changes and ongoing deforestation of the Atlantic Forest.

## 1 Introduction

Ancient and recent environmental and climatic changes shape the evolutionary trajectories of natural populations, leaving lasting genomic signatures. Changes in population demography, such as population decline and isolation, directly impact genetic diversity, inbreeding levels, and genetic load. As populations shrink or become fragmented, both inbreeding levels and genetic drift increase, leading to a loss of genetic variability and making species more vulnerable to genomic erosion and extinction (Pinto et al. 2024). Genomic erosion refers to the harmful genomic imprints that threaten small populations, including genome‐wide diversity loss, increase of expressed genetic load, and maladaptation (Dussex et al. 2023). There has been a recent surge in genomic studies of small and threatened populations, leveraging whole‐genome data to estimate multiple metrics of genomic erosion. This holds great promise for advancing conservation genomics by helping to identify vulnerable populations and design targeted conservation strategies to ensure long‐term species viability (Bosse and van Loon 2022). However, such efforts remain largely underrepresented for Neotropical species (Vilaça et al. 2024), emphasising the need for broader taxonomic and geographic sampling to better understand the interconnections between environmental changes and genetic health.

Maned Three‐toed Sloths (Xenarthra: Bradypodidae), the only xenarthrans endemic to Brazil's Atlantic Forest, are characterised by a black mane on the hindneck of the adults (Hayssen 2009). Like other sloths, they are strictly arboreal and have low metabolic rates due to their diet composed of leaves and shoots, causing them to remain immobile in trees for extended periods (Chiarello 1998). As a result, they are difficult to observe in the dense forest canopy, contributing to limited knowledge about their biology (Chiarello 2008). Additionally, their behavioural traits and arboreal habits make them highly vulnerable to deforestation and fires.

The Maned Three‐Toed Sloths' habitat is facing alarming conservation threats. Despite being one of the most important biodiversity hotspots Mittermeier et al. 2011, the Atlantic Forest has suffered extensive deforestation due to anthropogenic activities. Only about 8% of its original extent remains (Brown et al. 2020), with the current landscapes mostly composed of small and fragmented patches surrounded by open habitats, such as pastures and agricultural fields (Ribeiro et al. 2009; Vancine et al. 2024). However, restoration projects implemented over the last years are responsible for an increase in the forest cover in specific areas (Romanelli, 2022). These new forest formations are crucial for biodiversity conservation, especially for sloths, which depend on the presence of high and connected canopies for their survival. The challenges posed by habitat fragmentation underscore the need to evaluate its impact on the genetic health of sloth populations, which can offer valuable insights to inform more effective conservation strategies.

In addition to the recent anthropogenic environmental changes affecting sloth conservation, the Atlantic Forest has also experienced significant historical changes that have influenced the distribution and abundance of its biodiversity. During the climatic oscillations of the Quaternary, the persistence of stable green areas, or refugia, varied across the Atlantic Forest, significantly impacting the spatial patterns of biodiversity and endemism (Carnaval and Moritz 2008; Peres et al. 2020). The Maned Three‐toed Sloths also appear to be affected by this, as the remaining populations exhibit deep genetic divergence that has accumulated over thousands of years (Lara‐Ruiz et al. 2008; Schetino et al. 2017). Genomic imprints can reveal how these species have adapted to past climatic oscillations, offering valuable insights into their evolutionary history and conservation needs.

Until recently considered a single species, Maned Three‐Toed Sloths have been subdivided through a comprehensive integrative taxonomic review (Miranda et al. 2022) into two distinct species: the Northern Maned Sloth ( *Bradypus torquatus* Illiger, 1811) inhabiting the states of Bahia and Sergipe, and the Southern Maned Sloth ( *Bradypus crinitus* Gray, 1850) found in the states of Espírito Santo and Rio de Janeiro. Although there are no differences in pelage, size, and body mass, the northern and southern lineages present distinct craniomandibular characters and diverged around 4.2 million years ago (mya), as estimated by coalescent species delimitation analyses using four mitochondrial and nuclear DNA markers. The authors recommended that, along with the taxonomic review, the conservation status of these species should be reassessed to effectively address the conservation challenges of each lineage. An extensive study of the genome‐wide diversity and divergence of the Maned Three‐Toed Sloths can greatly enhance conservation efforts by providing insights into the unique genetic characteristics and evolutionary history of the northern and southern lineages.

Our research leverages whole‐genome resequencing data compared against a chromosome‐level reference genome recently assembled by our group (Bein et al. 2024) to unravel genomic signatures in Maned Three‐Toed Sloths in the context of historical and contemporary environmental and climatic changes in the Atlantic Forest. We test whether genomic metrics—such as genetic diversity, inbreeding levels, genetic load, and historical population sizes—reveal signs of genomic erosion in Maned Three‐Toed Sloths, particularly given the extensive fragmentation of the Atlantic Forest, which has occurred both historically and, more acutely, in recent times (Santos et al. 2024). Our study is one of the first to demonstrate the power of high‐resolution genomic data in revealing the impact of changes to the habitat and environment on the genome‐wide diversity of endemic species across the Atlantic Forest. This study presents the framework for other species with similar demographic and ecological traits as the Maned Three‐Toed Sloth in such Neotropical ecosystems.

## 2 Materials and Methods

### 2.1 Sampling

**FIGURE 1:**
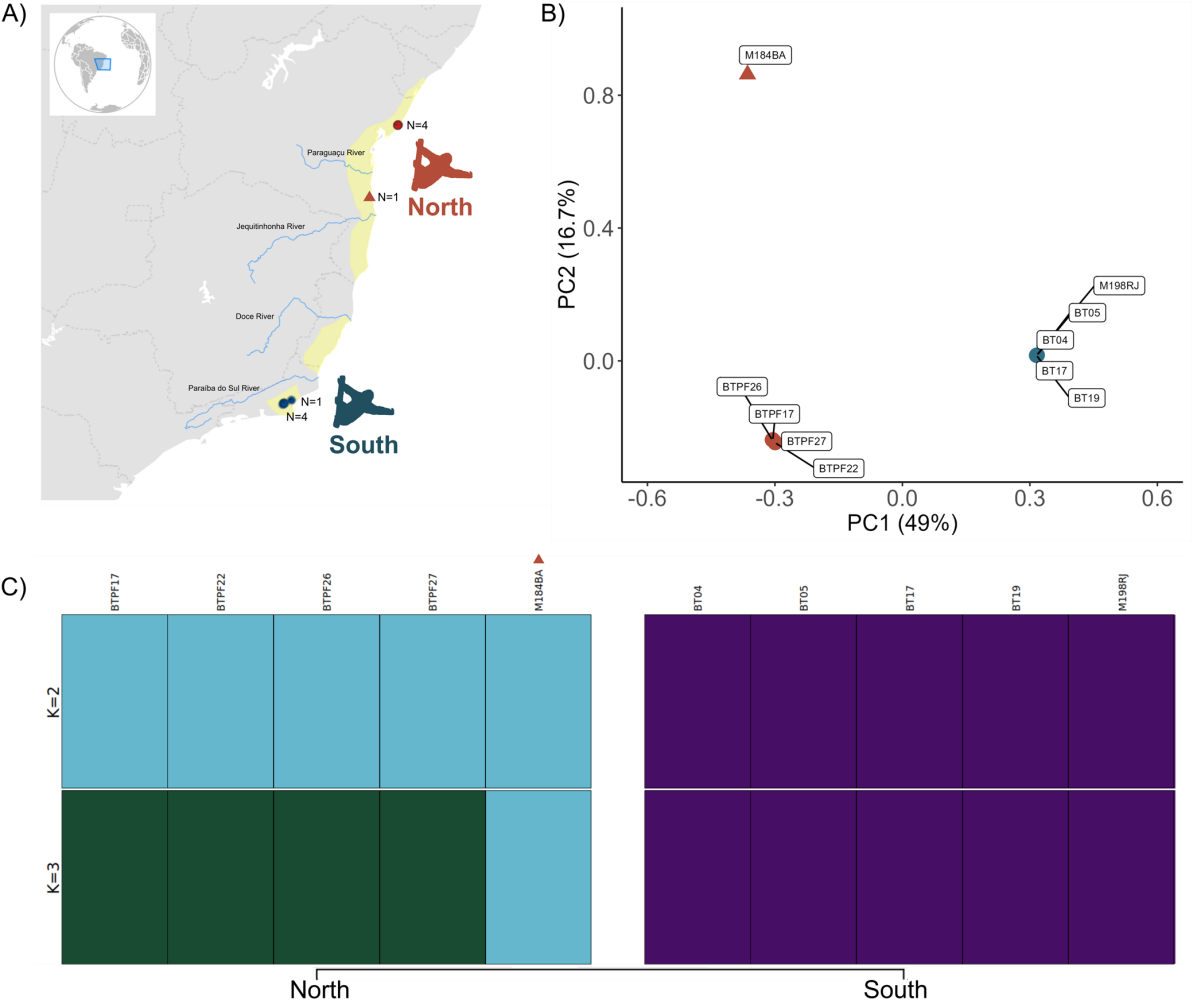
Genetic differentiation between the Northern and Southern Maned Sloths. (A) Map showing sample locations for Maned Three‐Toed Sloths throughout their distribution. The single individual from the Una population is represented by a triangle. (B) Principal component analysis with its respective variance explanatory power. (C) ADMIXTURE results showing the assignment probabilities of individuals (represented by columns) to genetic clusters (*K* = 2 and 3). The *K* = 3 represents the smallest cross‐validation error.

Blood or liver tissue samples from ten Maned Three‐Toed Sloths, five from each species, were collected in Atlantic Forest fragments in Brazil. Four Northern Maned Sloths were sampled in Mata de São João and one individual was collected in Una, both locations in Bahia state (Figure 1A). Four Southern Maned Sloths were originally sampled in Silva Jardim and one individual was collected in Rio das Ostras, both locations in Rio de Janeiro state. The collection date, sex, age, and geographical coordinates can be found in Table S1. Blood and tissue samples were stored in absolute ethanol at −20°C. The DNA was extracted using the DNeasy Blood and Tissue Kit (Qiagen). Samples were provided by the Centro de Coleções Taxonômicas da Universidade Federal de Minas Gerais and in the Museu Nacional da Universidade Federal do Rio de Janeiro and were exported under the licences CITES 138261 and ADD57E7, and SISGEN AF86294.

### 2.2 Library Construction

The 350 bp insert DNA library was prepared by using the NEB Next Ultra DNA Library Prep Kit. The genomic DNA was randomly sheared into short fragments by mechanical fragmentation using a Covaris sonicator. The obtained fragments were end‐repaired, A‐tailed, and further ligated with an Illumina adapter. The adapter‐ligated fragments were PCR amplified, size selected, and purified. The library underwent quantification using Qubit and real‐time PCR, while its size distribution was detected using the Bioanalyzer. The libraries were pooled, and paired‐end 150 bp reads were sequenced on the Novaseq 6000 Illumina platform.

### 2.3 Preprocessing Analysis and SNP Calling

Initially, the sequenced reads underwent Illumina adapter trimming using Cutadapt. Then, reads were filtered by minimum Phred quality score 30 with Trimmomatic (Bolger et al. 2014). Subsequent steps were run using the pipeline jATG (https://github.com/diegomics/jATG/tree/devel). Briefly, the filtered reads were mapped against the high‐quality chromosome‐level genome assembly of the Southern Maned Sloth (GCA_963992745) using BWA‐MEM v2.2.1 (Vasimuddin et al. 2019). Short and long read data derived from the Southern Maned Sloth reference genome sample were not used in the analyses, as the sample contains a mixture of wild individuals (Bein et al. 2024). PCR duplicates from BAM files were identified using MarkDuplicates (GATK). SNP calling was performed using GATK (v4.2.6.1) HaplotypeCaller, followed by GenotypeGVCF. Only positions on autosomal scaffolds longer than 5 Mbp were considered in the downstream analysis, as they should represent all full chromosomes, except for X and Y. SNPs located in masked regions, identified by analyzing the genome with Dfam TE Tools (v1.85) using RepeatModeler (Flynn et al. 2020) and RepeatMasker (Smit et al. 2015), were excluded from subsequent analyses. Furthermore, variants were filtered using BCFtools (Danecek et al. 2021) with GATK's recommended parameter thresholds (Table S2—Van der Auwera and O'Connor 2020). Additionally, SNPs with depth lower than 8× and greater than 2× the average coverage were also removed from subsequent analyses. All filtered genotypes were turned into missing data, producing a base‐pair resolution gVCF file.

### 2.4 Genetic Diversity and Inbreeding Analyses

**FIGURE 2:**
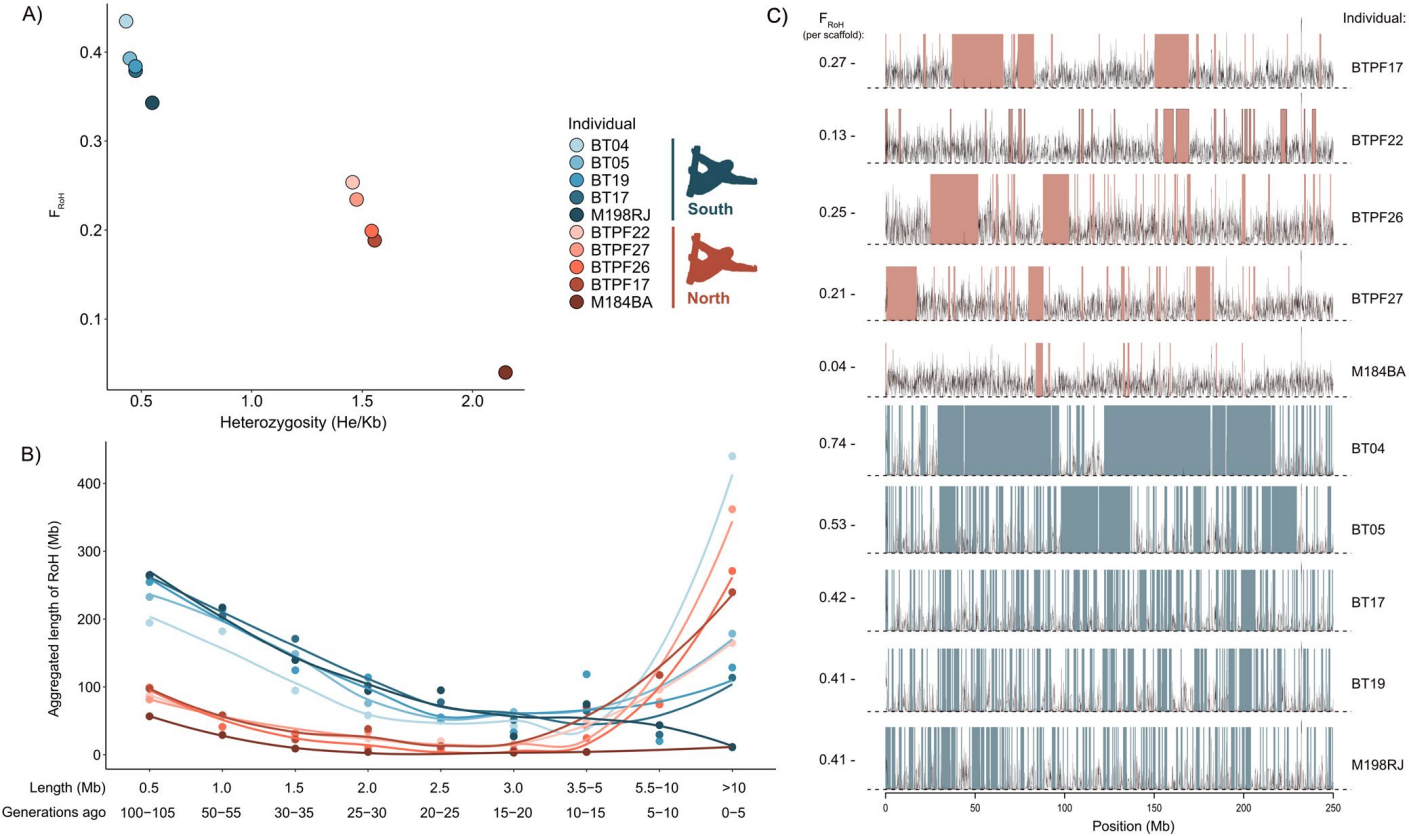
Genomic diversity and inbreeding levels (calculated as the fraction of the genome in runs of homozygosity—*F*_RoH_) for all sequenced individuals of Maned Three‐Toed Sloths. (A) Average genome‐wide heterozygosity (He) versus *F*_RoH_, showing a strong and significant negative correlation (Pearson correlation coefficient: *R* = −0.9745, *p* < 0.01). (B) Distribution of aggregated lengths of RoH (in Mb) in intervals of five generations and its associated expected number of generations since the individual's maternal and paternal lineages shared a common ancestor for different genome sections. The line represents a regression (LOESS) fit, capturing the underlying trend in the data. (C) Variation of genome‐wide heterozygosity across the scaffold one, depicting He of non‐overlapping 50 kb windows with RoH segments highlighted. Values in the *y*‐axis represent the chromosome‐specific *F*_RoH_ values.

Aiming to explore the genome‐wide patterns of genetic diversity, we estimated heterozygosity and performed runs of homozygosity (RoH) analysis using Darwindow (de Jong et al. 2023). This tool enables the assessment of RoH calls accuracy through visual inspection of heterozygosity and RoHs along scaffolds. Heterozygosity was calculated using a sliding‐window approach with non‐overlapping fixed‐length windows of 50 kb. For RoH detection, a window was classified as having low heterozygosity if its value fell below one‐fifth of the individual's mean heterozygosity. To define a RoH, a minimum of 10 consecutive 50‐kb windows with a maximum of 0.7 missing data per window was required. This final set of parameters was chosen by visually checking the fit of the RoH and heterozygosity, as demonstrated in Figure 2C. Furthermore, RoHs shorter than 500 kb were interpreted as indicative of background relatedness rather than recent consanguineous mating (Ceballos et al. 2018). The inbreeding level (*F*_RoH_) was calculated as the proportion of the genome marked as RoH.

Inbreeding results were also analyzed through an alternative RoH approach implemented in PLINK v2.0 (Chang et al. 2015), applying the same minimum size threshold of 500 kb as above, with the following parameters: minimum SNP count per RoH of 100, sliding window size of 100 SNPs, maximum number of heterozygotes per window of 1, and maximum number of missing sites per window of 15.

To estimate the time in generations since an individual's maternal and paternal lineages shared a common ancestor, we converted the lengths of RoH tracts to generations using an estimated average mammalian recombination rate of 1 cM/Mb and the equation *g* = 100/(2*rL*), where *g* is the time in generations, *r* is the recombination rate, and *L* is the length of the RoH tract in Mb (Kardos et al. 2018; Saremi et al. 2019). Additionally, the kinship coefficients between individuals were estimated with PLINK v2.0.

### 2.5 Population Structure

To investigate the population structure and admixture patterns between Maned Three‐Toed Sloths lineages, we employed ADMIXTURE and Principal Component Analysis (PCA). A dataset suitable for robust population structure analysis was generated by applying filtering criteria to retain unlinked SNPs present in the majority of individuals, minimizing biases introduced by missing data and linkage disequilibrium (LD). Specifically, we filtered biallelic SNPs with a maximum missing rate of 0.8 and performed LD pruning using PLINK v2.0 with the following parameters: –indep‐pairwise 20 20 0.5. PCA was performed using PLINK v2.0. Assignment probabilities of individuals to genetic clusters (*K*) were calculated with ADMIXTURE (Alexander et al. 2009) implemented in Admixpipe (Mussmann et al. 2023) testing *K* from 1 to 6, with each *K* value evaluated with 10 replicates run in parallel. The best‐fit number of *K*, which maximized classification in an ADMIXTURE cross‐validation process, was selected with 20% cross‐validation. Results were clustered and visualized with CLUMPAK (Kopelman et al. 2015).

### 2.6 Demographic Analysis

Demographic trajectories in different time scales were inferred using the Multiple Sequentially Markovian Coalescent method MSMC2 (Schiffels and Wang 2020). This method infers population size history by analysing the distribution of times to the most recent common ancestor (TMRCA), estimated from genetic diversity levels across the genome. The only Northern Maned Sloth sample from Una was excluded because our analysis revealed that it is genetically differentiated from the other Northern Maned Sloths (https://paperpile.com/c/4uiyGk/QfIF+4Z46) (Figure 1). This decision was taken to avoid introducing bias related to population structure (Hilgers et al. 2024; Mather et al. 2020). Thus, to enhance statistical power, demographic analysis was performed by combining five samples from the southern lineage and four samples from the northern lineage. Input files to run MSMC2 were prepared using scripts available at the MSMC Tools package (https://github.com/stschiff/msmc‐tools). First, the filtered gVCF files were converted into individual masks (indicating regions where each individual was called) using the VCFAllSiteParser.py script. Sexual and unlocalized scaffolds were removed from the gVCF file, as they can affect the demographic history inference (Gower et al. 2018). Subsequently, the MSMC2 input files were generated using the generate_multihetsep.py script. Then, bootstrap samples (*n* = 50) were generated using the multihetsep_bootstrap.py script (−*n* 50 –s‐20000000–chunks_per_chromosome 10). MSMC2 was run with the default pattern of fixed time segments (1 × 2 + 25 × 1 + 1 × 2 + 1 × 3) using individual masks and the mappability mask for each chromosome in order to focus on regions of the genome with sufficiently high mappability (i.e., no repeat regions and other complex features). *N*_*e*_ trajectories were scaled by setting the per generation mutation rate to 7.97 × 10^−9^, the average mammalian mutation rate estimated by Bergeron et al. 2023, and the generation time to 3.9 for Maned Three‐Toed Sloths (Pacifici et al. 2013). This mutation rate was selected due to the lack of specific estimates for sloths or closely related species. It is important to note that while the selected mutation rate and generation time influence the scale of the horizontal axis and the estimate of the effective population size, they do not affect the qualitative shape of the demographic curve estimated by SMC analysis (Mather et al. 2020).

### 2.7 Phylogenomic Reconstruction

The estimation of the divergence time between Northern and Southern Maned Sloths was performed using the software SNAPP implemented on BEAST v2.6.4 (Bouckaert et al. 2014). One randomly selected individual from each of the two main genetic lineages of the Maned Three‐Toed Sloths, along with the single individual from the Una population, was included in the analysis, as coalescent analysis does not require a large number of individuals and has a high computational cost (Stange et al. 2018). The species Linnaeus's two‐toed sloth ( *Choloepus didactylus* —Genbank accession number GCF_015220235) and brown‐throated sloth ( *Bradypus variegatus* —GCA_004027775) were chosen as outgroups. The filtered reads of all genomes were mapped against the *C. didactylus* genome and the SNP calling was performed as described previously. The resulting VCF file was strictly filtered to include only bi‐allelic SNPs with no missing data in the autosomal repeat‐masked scaffolds and to remove SNPs under linkage disequilibrium (vcftools thin option = 100,000), reducing the dataset to 23,894 SNPs. The SNAPP input file was prepared using the script snapp_prep.rb (https://github.com/mmatschiner/snapp_prep) (Bryant et al. 2012). The genus *Bradypus* was constrained to be monophyletic and one calibration point based on a fossil record for the Folivora node was set to 15.97–40.6 mya with a uniform distribution (Vizcaíano and Scillato‐Yané 1995). Two independent runs were performed with 10 million Monte Carlo Markov Chain (MCMC) generations each, sampling every 5000 generations. Convergence between runs and stationarity of the ESS (effective sample sizes) values were checked with Tracer v.1.7 and required to be higher than 200 (Rambaut et al. 2018). All trees from the two independent runs (with 10% burn‐in) were combined with LogCombiner v.2.4.5 and a maximum clade credibility tree was obtained calculating mean heights in TreeAnnotator v.2.4.5 (Helfrich et al. 2018).

### 2.8 Genetic Load

We compared the genetic load between the Northern and Southern Maned Sloths to assess potential differences in their genomic health. The software SnpEff (Cingolani et al. 2012) was used as a tool to annotate and predict the consequence of genetic variation on genes and proteins. First, a database in SnpEff was created using the *Bradypus crinitus* genome annotation obtained with TOGA (Tool to infer Orthologs from Genome Alignments) (Kirilenko et al. 2023), a tool that infers orthologous genes by projecting annotations from selected species (https://genome.senckenberg.de/download/TOGA). Considering that most of the deleterious mutations are likely to be derived alleles, the ancestral state of each site in the Southern Maned Sloth reference genome was inferred using the Hoffmann's two‐toed sloth ( *Choloepus hoffmanni* —GCA_000164785), nine‐banded armadillo ( *Dasypus novemcinctus* —GCF_030445035), and Southern tamandua ( *Tamandua tetradactyla* —GCA_023851605) as outgroup species. The masked genomes were converted into fastq reads by sliding across the genome in windows of 70 bp starting every 10 bp (so reads overlap by 60 bp) and transforming each window into a fastq read. These reads were mapped against the Southern Maned Sloth reference genome with BWA‐MEM v.0.7.17 with default parameters. The resulting bam files for the three outgroup species were merged, and the consensus alignment was extracted using ANGSD 0.940 (–doFasta 2 –doCounts 1) (Korneliussen et al. 2014). Then, the SNPs in the sloths VCF were polarised based on the alleles in the consensus alignment using PLINK v2.0. Only positions present in the consensus sequence were kept in the final VCF.

Using the polarised VCF with a maximum missing rate of 0.8, each SNP was annotated according to the functional effect prediction using SnpEff v. 5.2, and the number of high (loss of function—LoF) and moderate (missense) impact mutations was calculated. The sites with warnings were removed from the VCF, as they indicate issues in the reference genome that could lead to incorrect variant annotations. We measured three proxies of genetic load. First, the number of heterozygous genotypes was counted as a proxy for masked genetic load, representing deleterious alleles that are partially hidden from selection but can potentially be expressed in future generations. Second, the number of homozygous genotypes for the derived allele (hereafter referred to as homozygous‐derived) was counted as a proxy for realised genetic load, reflecting deleterious alleles with effects on the current generation. Third, the total number of derived deleterious alleles was calculated as the number of homozygous‐derived genotypes multiplied by two, as homozygous alleles are represented twice, plus the number of heterozygous genotypes. This measure accounts for a scenario where deleterious mutations exhibit an approximately additive effect. Mann–Whitney *U* tests were used to test for differences in the number of deleterious mutations between the groups.

Aiming to perform a robust lineage comparison accounting for potential reference bias, *R*_*xy*_ statistics were performed as described in Do et al. (2015) and implemented by Xue et al. (2015). This method estimates the relative number of derived alleles found at sites in one lineage rather than the other, for a particular variant category, such as LoF and missense. The ratio is normalized based on the accumulation of mutations at sites expected to evolve neutrally (Do et al. 2015), thereby reducing population‐specific biases, such as mapping bias introduced by using a Southern Maned Sloth reference genome. To achieve this, we assumed that intergenic sites represent putatively neutral regions and extracted a random subset of 100,000 intergenic SNPs to standardize the estimates. To confirm the efficiency of the polarization method, we also compared the number of derived alleles in intergenic sites for a non‐polarized dataset. *R*_*xy*_ equal to 1 indicates no change in frequency between two populations, while *R*_*xy*_ < 1 indicates a decrease and *R*_*xy*_ > 1 an increase in frequency in population *x* relative to population *y*. The sampling error (95% bootstrap confidence intervals) was calculated by randomly resampling SNPs with replacement 100 times and recalculating *R*_*xy*_.

Additionally, the relation between *F*_RoH_ and the number of homozygous‐derived and heterozygous genotypes was tested using a linear regression model.

## 3 Results

### 3.1 Variant Calling

Whole‐genome resequencing data was obtained for ten Maned Three‐Toed Sloths, five from the Northern and five from the Southern Maned Sloth (Figure 1A and Table S1). The average genome coverage per individual was 21.4 ± 2.7. The number of variants for each individual is presented in the Table S1. After filtering by biallelic positions, a maximum missing rate of 80%, and linkage disequilibrium, a set of 2,845,496 SNPs was obtained and used in the population structure analysis. Kinship analysis showed no close relationship (first‐ or second‐degree relations) between the analysed individuals.

### 3.2 Genetic Divergence and Diversity

Population structure analysis confirmed clear genetic differentiation between the Southern and Northern Maned Sloths, as evidenced by the ADMIXTURE and PCA results (Figure 1). The first principal component (PC) separated northern from southern lineages, explaining 49% of the variance, and the second PC separated one individual from Una, a southern location in Bahia state, from individuals from Mata de São João (MST), the northern location in Bahia, explaining 16.7% of the variance. Assignment probabilities of individuals to genetic clusters performed by ADMIXTURE showed a similar geographic pattern, supporting three genetic clusters with no shared ancestry proportion (Figure S1).

The average genome‐wide heterozygosity is higher in the Northern Maned Sloth compared to the Southern Maned Sloth. Consistently, the overall inbreeding level, estimated as the proportion of the genome in runs of homozygosity (*F*_RoH_), is lower in the northern than in the southern population (Figure 2A). RoH tract lengths were converted to generations, providing estimates of the time since the individual's maternal and paternal lineages shared a common ancestor. For instance, RoH longer than 10 Mb indicate inbreeding events in the last 5 generations (Figure 2B). This analysis reveals that the southern population exhibits a higher number of short RoH (i.e., older inbreeding) compared to the northern. However, Darwindow results showed a recent increase in inbreeding levels in the northern lineage over the last 10–15 generations, which has reversed the historical pattern. This shift has led to higher levels of inbreeding in the north compared to the south over the last 5 generations. In contrast, individuals from the southern population have experienced varied ancestry histories. For example, individuals BT17 and BT19 exhibit a stable inbreeding trend, with a slight decrease in inbreeding levels over the last 5–10 generations. In a distinct trend, BT04 appears to have closely related ancestors in a very recent timeframe, approximately 5 generations ago. Meanwhile, M198RJ shows a decrease in inbreeding levels over the last 5 generations. Eight out of ten individuals were sampled in a similar timeframe (2019–2021), while the other two individuals were sampled in 2003 (Table S1). Interestingly, the two oldest individuals from the early 2000s exhibit the lowest inbreeding levels within their lineage.

Inbreeding levels over time, as estimated with an alternative approach implemented in PLINK, display a shift pattern similar to those observed with Darwindow, with an increase in the aggregated length of RoH in the last generations in the northern lineage and a stable or slight decrease in the southern lineage (except for one individual) (Figure S2). However, the increase in the inbreeding level for the northern lineage begins earlier in PLINK results, around the last 50 generations. This discrepancy likely arises from differences in the sliding window approaches and parameter settings between Darwindow and PLINK methods, with PLINK being more restrictive in identifying long runs, which were visually confirmed in Darwindow (Figure 2C).

### 3.3 Divergence Time Between Species

**FIGURE 3:**
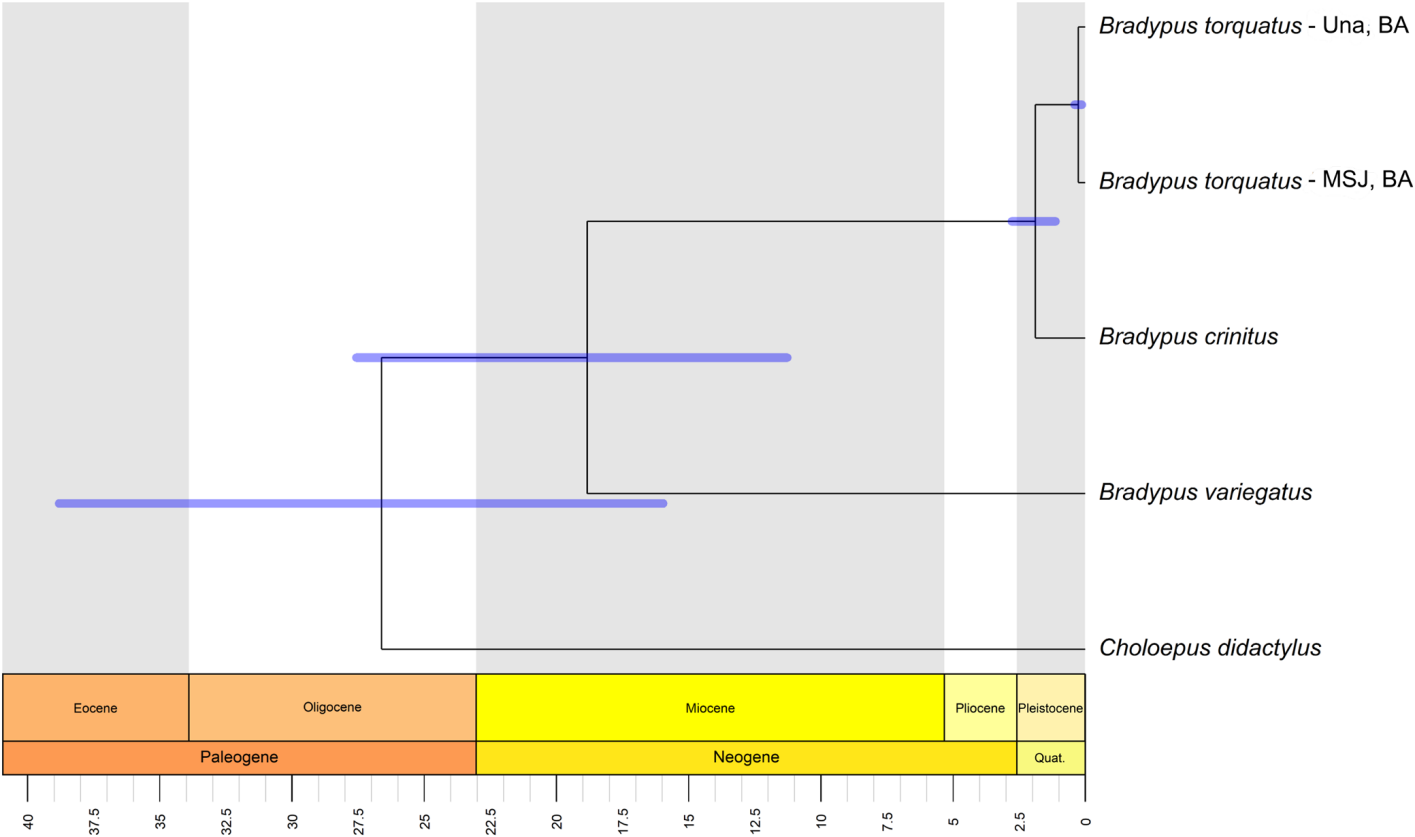
Estimates of divergence times among major Maned Three‐Toed Sloths clades included in this study. Divergence times and 95% highest posterior density (HPD) intervals were estimated using a set of 23,894 autosomal SNPs in SNAPP. Linnaeus's two‐toed sloth ( *Choloepus didactylus* ) and brown‐throated sloth ( *Bradypus variegatus* ) were included as outgroups. Una and MST (Mata de São João) are two localities in Bahia, where the *Bradypus torquatus* individuals were sampled.

Given the genetic differentiation between the northern and southern lineages observed in the population structure analysis, we next sought to determine the timing of their divergence. The phylogenetic analysis performed using autosomal SNPs showed that the individuals from the cryptic new species formed well‐supported and separate monophyletic lineages (SNAPP posterior probabilities = 1.0). The northern and southern lineages diverged at 1.88 mya (95% highest posterior density [HPD] = 1.13 to 2.78 mya) (Figure 3). The individual from Una diverged from the population from Mata de São João (MST) at 0.266 mya (95% HPD = 0.14 to 0.41).

### 3.4 Ancient Demographic History

**FIGURE 4:**
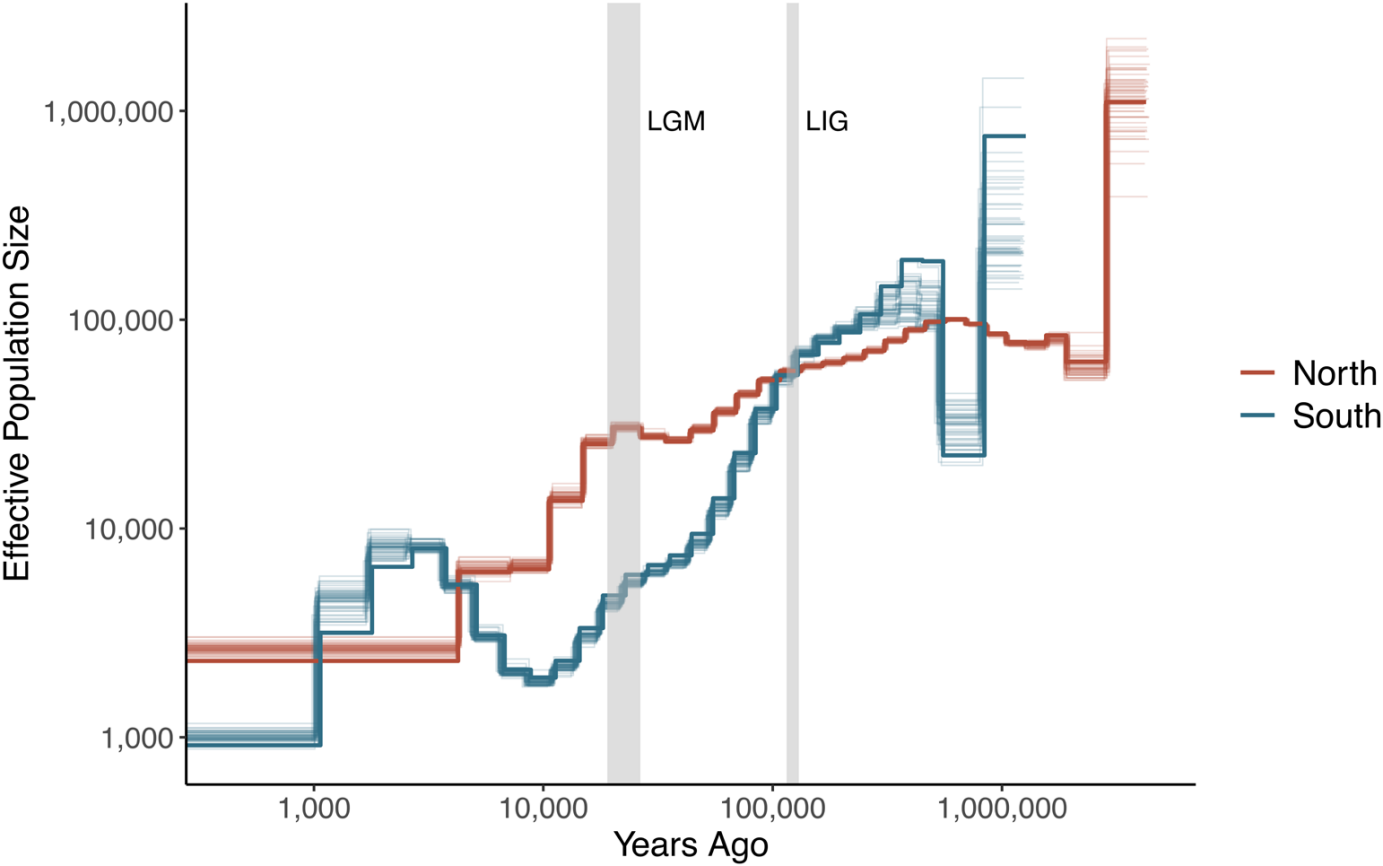
Historical demographic trajectories of Northern (red) and Southern (blue) Maned Sloths reconstructed using the Multiple Sequentially Markovian Coalescent (MSMC) model (https://paperpile.com/b/4uiyGk/NXgB). Bootstrap replicates (50 for each lineage) are plotted in lighter lines. Shaded lines indicate the Last Glacial Maximum (LGM) and the Last Interglacial (LIG) period. Inferred fluctuations in effective population size (*N*_*e*_) were rescaled using 3.9‐year generation time and 7.97 × 10^−9^ per generation mutation rate assumptions.

Considering the long‐term independent evolutionary history of the Southern and Northern Maned Sloths, we hypothesize that they may also exhibit distinct demographic histories. We tested this by estimating changes in effective population size (*N*_*e*_) over time using the Multiple Sequentially Markovian Coalescent method MSMC2. This analysis was performed for each lineage by combining four and five individuals from the Northern and Southern Maned Sloths, respectively. The only individual from Una was excluded, as this represents a unique individual from a distinct genetic pool, and the population structure can be misinterpreted as population expansion by MSMC2 (Hilgers et al. 2024; Mather et al. 2020). The results showed that the Northern and Southern Maned Sloths presented a population decline starting approximately 500,000 years ago, during the early Pleistocene (Figure 4). The southern lineage presented a more drastic decline, followed by an expansion after the Last Glacial Maximum (LGM), which was not observed in the northern lineage.

### 3.5 Genetic Load

**FIGURE 5:**
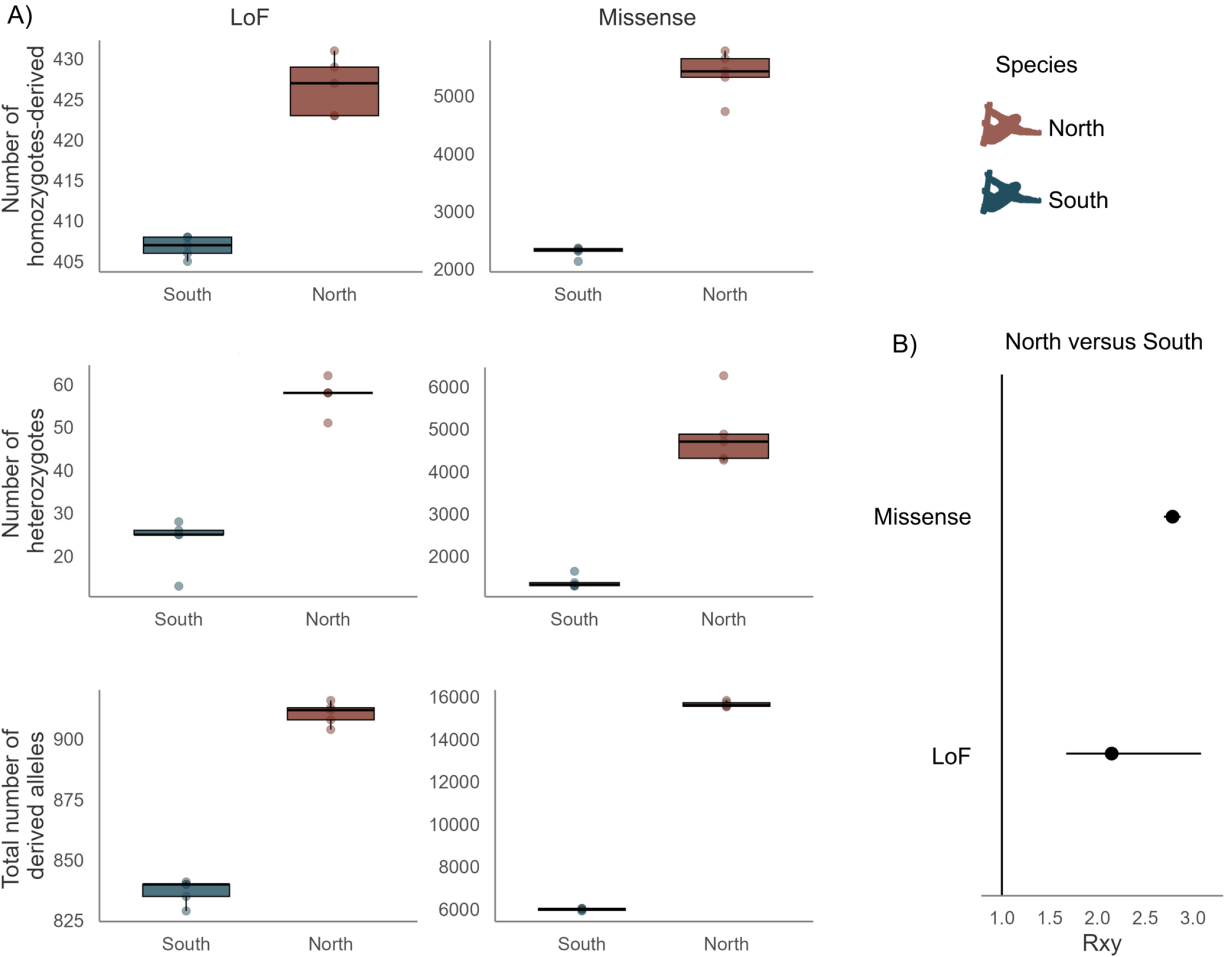
(A) Number of homozygous‐derived and heterozygous genotypes and total number of derived alleles per individual for missense and loss‐of‐function (LoF) mutations for the Southern and Northern Maned Sloths, (B) *R*_*xy*_ statistics showing a relative frequency excess of missense and LoF mutations in the Northern in relation to the Southern Maned Sloths (*R*_*xy*_ > 1). Error bars represent 95% bootstrap confidence intervals.

We next asked whether the distinct demographic histories of the Northern and Southern Maned Sloths were reflected in differences in genetic load, using metrics derived from putative deleterious variants identified through an annotation‐based method. We calculated the number of high (loss of function—LoF) and moderate (missense) impact mutations. For each category, the number of homozygous‐derived and heterozygous genotypes was analysed separately, as a measurement of realised and masked genetic load, respectively (Bertorelle et al. 2022). This assumption presumes that most deleterious alleles are recessive, thereby being partially hidden from selection when in a heterozygous state. Consequently, homozygous‐derived genotypes most likely have an impact on the fitness of the current generation (realised load), while deleterious mutations in heterozygous genotypes are masked but can potentially be expressed in future generations (masked load) (Bertorelle et al. 2022). We also counted the total number of derived deleterious alleles, to address the possibility where deleterious alleles exhibit an additive effect. Our results showed that the Northern Maned Sloths present a greater absolute number of homozygous‐derived and heterozygous genotypes and total derived deleterious alleles than the Southern Maned Sloths (Figure 5A).

For a robust comparison between groups accounting for the potential reference bias, we estimated the ratio of derived alleles (*R*_*xy*_) between the Southern and Northern Maned Sloths for LoF and missense categories. This method standardises the comparison by using the number of derived alleles in intergenic regions. First, to check if this category really represents neutral variants, we calculated the total number of derived deleterious alleles in the intergenic regions. No significant difference in the number of derived alleles was observed between the northern and southern lineages (Figure S3A, Mann–Whitney *U*, *p* = 0.6905). When we performed the same comparison without redefining the ancestral state of each site in the VCF file based on the consensus allele determined from the outgroup species (non‐polarised dataset), the Northern Maned Sloths presented a higher number of derived alleles compared to the Southern Maned Sloths (Figure S3B). This result indicates that the polarisation strategy efficiently reduced the population bias introduced by using a Southern Maned Sloth reference genome. Using the polarised dataset (thereby minimising reference bias), *R*_*xy*_ statistics showed that there is a relative excess of missense and LoF mutations in the Northern in relation to the Southern Maned Sloths (Figure 5B).

Within each lineage, a positive correlation between inbreeding and the number of homozygous‐derived genotypes was observed for both missense and LoF mutations (Figure S4). Conversely, a negative correlation was observed between inbreeding and the number of heterozygous missense and LoF mutations. Some correlations were weak and non‐significant (Figure S4), likely due to the low sample size. These results are consistent with expectations: increased inbreeding reduces heterozygosity and increases homozygosity, thereby leading to a higher number of homozygous‐derived genotypes and a lower number of heterozygous genotypes for deleterious mutations such as missense and LoF variants.

## 4 Discussion

The analysis of the evolutionary history, demographic patterns, and comparative assessment of genetic diversity, inbreeding levels, and genetic load between populations of the Southern ( *B. crinitus* ) and Northern ( *B. torquatus* ) Maned Three‐Toed Sloths provided valuable insights into the evolutionary dynamics and conservation implications for these endangered species. Our study showed that these two lineages diverged in the early Pleistocene and have experienced different demographic histories. The Southern Maned Sloth has historically displayed a smaller population size, which likely led to lower genetic diversity and higher overall inbreeding levels compared to the Northern Maned Sloth. However, the northern lineage has experienced a recent increase in inbreeding levels in the last few decades and presents higher masked and realised genetic load. Together, these results reinforce the independent evolutionary paths of these two lineages and underscore the conservation challenges faced due to historical evolutionary events and recent deforestation of the Atlantic Forest. In particular, if inbreeding among Northern Maned Sloths continues, the accumulation of genetic load could cause inbreeding depression, raising the risk of local extinction.

### 4.1 Pleistocene Effects on Divergence, Genetic Diversity, and Demography of Maned Three‐Toed Sloths

Our results showed that the northern and southern lineages diverged in the early Pleistocene, around 1.88 mya (HPD = 1.13–2.78 mya). This is in accordance with previous estimates of Lara‐Ruiz et al. (2008), which were based on mitochondrial genes. However, previous studies using one mitochondrial and three nuclear genes estimated an older divergence time of approximately 4.24 mya (HPD = 1.59–6.74) (Miranda et al. 2022; Schetino et al. 2017). We consider our divergence time estimates more reliable because they are based on a dataset of multiple independently segregating loci, which better reflect true evolutionary relationships between populations. Thus, our estimates generally fall within the confidence intervals of previous studies (Miranda et al. 2022; Schetino et al. 2017), while exhibiting narrower times, thereby improving upon earlier results by offering greater precision.

Divergence between northern and southern lineages is most likely associated with vicariant events promoted by climatic oscillations and geographic barriers. Specifically, the Doce river valley is known as an important turnover (spatial changes in species composition) for several taxa (Peres et al. 2020), most likely linked to climatic differences between the northern and southern regions of the valley, rather than to the river itself (Saiter et al. 2016). Forest contraction and expansion associated with glacial cycles in the Pleistocene led to the isolation of populations, which subsequently accumulated genetic differences (Carnaval and Moritz 2008). As sloths are strictly arboreal, they are especially vulnerable to fragmentation and discontinuity of forest habitats.

Our results also showed that the single individual from Una diverged from the northern population in Bahia (MST) around 0.266 mya (95% HPD = 0.14–0.41). These two locations are separated by the Paraguaçu River, which has also been shown to be a barrier for other species (Cazé et al. 2016; Pellegrino et al. 2005). An additional important population structure pattern observed for the Maned Three‐Toed Sloths occurs in the south. Rio de Janeiro and Espírito Santo states present a break in the species distribution accompanied by genetic divergence (Lara‐Ruiz et al. 2008; Schetino et al. 2017). To provide a more comprehensive overview of the species' evolutionary history, representatives from Espírito Santo should be included, especially considering that there are significant phenotypical (and potential adaptive) differences associated with altitude in this location (Lara‐Ruiz and Chiarello 2005).

Northern and Southern Maned Sloths experienced a similar demographic trajectory, undergoing population decline since the early Pleistocene. However, the southern lineage experienced a more drastic decline. This result corroborates the refugia hypothesis in the Atlantic Forest through the Pleistocene demonstrated by Carnaval and Moritz (2008). The authors modelled the forest range in different climatic scenarios and showed that the northern and southern regions of the Doce River experienced strikingly different Quaternary histories. While the northern region exhibited large forest refuges, the south went through a relative instability of forest habitats. Consequently, the reduced habitat availability in the south during the forest contractions of the LGM restricted the populations to smaller sizes. This also explains the post‐LGM population expansion observed in the Southern Maned Sloth population analyzed, as forest expansions allowed colonisation of non‐refuge areas, a pattern already documented for other species (Batalha‐Filho and Miyaki 2016; Brunes et al. 2015; Cabanne et al. 2007). In contrast, the more stable and larger refuges available for the northern populations explain the higher genetic diversity observed for this lineage. Thus, the spatial variation in historical forest refugia in the Atlantic Forest is responsible for the current patterns of biodiversity and endemism observed along its distribution, with more stable areas presenting higher genetic diversity (Carnaval et al. 2009).

Genetic variation rises from random changes that are not only beneficial but mostly deleterious, potentially reducing the mean fitness of a population (Bertorelle et al. 2022). The Northern Maned Sloths presented a higher masked and realised genetic load compared to the southern lineage. While some studies have shown that small and isolated populations tend to have a high genetic load (Khan et al. 2021; Dussex et al. 2021), other research suggests that species with historically small population sizes and low genetic diversity may exhibit a lower genetic load compared to species with larger populations (van der Valk et al. 2019). Since we utilised the reference genome and annotation from the Southern Maned Sloth, we recognise that our predictions of the phenotypic impact of variants could be influenced by reference bias. For instance, this bias may have reduced the predicted number of LoF and missense variants in the southern lineage. We addressed the potential bias through two approaches: (1) redefining the ancestral state of each site based on the consensus allele from outgroup species, ensuring a more reliable evolutionary framework, and (2) applying the *R*_*xy*_ statistic, which standardises group comparisons by leveraging the number of derived alleles in intergenic regions (representing neutral variation). This analysis confirmed an excess of both LoF and missense mutations in the northern compared to the southern lineage, reinforcing the validity of our findings.

We hypothesize that the lower genetic load observed in the southern lineage is a result of its historically small population size, which has consistently experienced higher levels of inbreeding. Mating between related individuals likely provided an opportunity for previously hidden (masked) deleterious mutations to become expressed, increasing the realised load while decreasing the masked load (Bertorelle et al. 2022). Subsequently, the exposure of recessive deleterious alleles to purifying selection would have reduced the realised load over time (van der Valk et al. 2019). Recently, several studies have reported purging of genetic load, especially in long‐term isolated and inbred populations (Dussex et al. 2021; Grossen et al. 2020; Kleinman‐Ruiz et al. 2022; Robinson et al. 2018). This suggests that while the smaller population in the southern Atlantic Forest may have experienced a reduction in the relative number of highly deleterious mutations due to purifying selection, the northern populations may face a higher fitness cost associated with a greater burden of deleterious homozygous genotypes. Rapid population declines especially affect large populations with high genetic diversity such as the Northern Maned Sloths, as they carry many deleterious alleles that can reach fixation before genetic purging can remove them (van der Valk et al. 2019). We acknowledge that the results presented here should be interpreted with caution, as there are still gaps in understanding how measures of deleteriousness reflect the intensity of selection acting on polymorphisms or how reduced genetic load translates into fitness outcomes in species of conservation concern (Willi et al. 2022).

These findings underscore the complex interplay between population size, genetic diversity, and genetic load in shaping the genetic health and long‐term viability of endangered species. They emphasise the urgent need for targeted conservation efforts to mitigate further population declines and preserve genetic diversity in both Southern and Northern Maned Sloth populations. Improved conservation outcomes are more likely to be achieved when both maximizing genetic diversity and minimizing genetic load are considered simultaneously (Bertorelle et al. 2022).

### 4.2 Recent Conservation Challenges Faced by Maned Three‐Toed Sloths

The contrasting patterns of genetic diversity, inbreeding, and genetic load observed between the Northern and Southern Maned Sloth populations emphasize the distinct conservation challenges faced by these two lineages. The northern population exhibits higher genetic diversity and lower overall levels of inbreeding compared to the southern population. However, our results show increasing inbreeding levels over the past few decades for the northern lineage. Although pinpointing the exact onset of these increased inbreeding events is challenging, it most likely coincides with the escalating deforestation rates in the Northern Atlantic Forest in the last few decades. Livestock farming and ranching, wood and pulp plantations, and residential and commercial development have significantly intruded into and diminished sloth habitats, leading to population fragmentation and decreased connectivity (Santos et al. 2024) (Figure S5). In fact, deforestation in the Atlantic Forest has impacted many species, leading to extreme reductions in their distribution ranges, reaching up to 90.7% for some taxa (Brown et al. 2020). This scenario highlights the urgent need for targeted conservation efforts in northern Atlantic Forest regions, in particular in the state of Bahia, to mitigate genetic erosion and preserve the adaptive potential of the species in the face of ongoing environmental changes.

Given the rich biodiversity and genetic diversity found in the Northeast Atlantic Forest—an important refugial area for many species—conservation efforts should prioritise this region. However, Brazilian conservation units currently focus on the southern and southeastern regions, which are also under great threat, but carry lower diversity and are predominantly occupied by post‐LGM expansion taxa (Carnaval et al. 2009). It is crucial for Brazilian conservation strategies to recognise the importance of additionally protecting these key historical refugia areas.

Conversely, the low genetic diversity and high overall inbreeding levels observed for the Southern Maned Sloth are attributed to a smaller historical population size exacerbated by habitat loss and human activities in the last centuries. It shows that historical habitat loss (during glacial cycles) can continue to threaten populations in future generations by increasing inbreeding and decreasing diversity (Pinto et al. 2024). Despite the significant deforestation, the Southern Maned Sloth habitat has recently experienced forest gains that outweighed habitat loss (Santos et al. 2024). Specifically, four individuals analysed in this study were collected within or around the Poço das Antas Biological Reserve (PABR), a protected area established in 1974. This timeframe coincides with a slight decline or stability in inbreeding levels for three individuals, possibly facilitated by new connections among forest fragments due to successful habitat restoration efforts.

Recent population recovery in this protection area has been reported for other strictly arboreal species. The endangered golden lion tamarin has experienced a substantial population recovery, increasing from just 200 individuals in 1971 to 4800 by 2023 (Menegassi and Ferraz 2023; Ruiz‐Miranda et al. 2019). This remarkable turnaround is attributed to several conservation management actions promoted by the Golden Lion Tamarin Conservation Program and Golden Lion Tamarin Association since 1983 (Kierulff et al. 2012). These efforts have involved extensive reforestation work, including the restoration of 338 ha and the establishment of 25 native forest corridors to connect important forest fragments (Ruiz‐Miranda et al. 2019) (Figure S5C). Besides reforestation, it cannot be discarded that cases of reintroduction of individuals by local conservation programmes might also play a role in the distribution of the current genetic diversity of Maned Three‐Toed Sloths (Miranda, F.R., personal communication). Although we observed a slight decrease or a stable trend in inbreeding levels in only three individuals, overall, the inbreeding levels are not increasing as dramatically as observed in the northern population. Indeed, a single individual from the southern lineage exhibited evidence of substantial recent inbreeding, which shows that mating of related individuals is still happening and additional efforts for expanding the forests and connecting forest fragments are important. We acknowledge that the findings reported herein were based on a limited sample size. Expanding it would strengthen the analysis, particularly in accurately estimating the recent population size, given the demanding effort for field work due to the low detectability and high complexity involved in sampling wild sloths. Nevertheless, our results point to the success of conservation practices related to the establishing of protected areas and reforestation to safeguard the persistence of the species and maintenance of its critical genetic diversity and adaptive potential.

Notably, two individuals analysed in this study were sampled along the BR‐101 highway, which bisects the PABR. In addition to directly causing mortality for numerous individuals of several species (including one of the sequenced individuals here), this road also functions as a potential barrier to gene flow (Ascensão et al. 2019). Therefore, this issue demands urgent attention and effective action to safeguard the benefits of reforestation efforts in this region from being undermined by other anthropogenic disturbances.

The pronounced genetic divergence between northern and southern lineages emphasizes the necessity of reevaluating their conservation status. Currently listed as a single taxon in the IUCN red list under the vulnerable category (Chiarello et al. 2022), the validation of both species would reduce their ranges and may warrant listing in a more threatened category (Miranda et al. 2022; Santos et al. 2024). Our results support that each lineage has an independent evolutionary history and is facing unique conservation challenges, which should be addressed accordingly to guarantee the conservation of its entire genetic diversity. By integrating genetic insights into conservation practices, policymakers can make more informed decisions to improve management strategies and protect species.

### 4.3 Genomic Erosion and Conservation Implications in Fragmented Populations

Species are increasingly threatened by rapid environmental changes driven by human activities. Their capacity to adapt will determine whether they will persist or face extinction. Past and present demographic events leave genomic signatures, which can be assessed and used as a proxy to inform genetic health and guide conservation efforts (Díez‐Del‐Molino et al. 2018). This study has revealed distinct genomic erosion patterns experienced by two different lineages across different timescales.

Numerous studies have shown that habitat loss poses a persistent threat to wildlife populations by triggering genomic erosion (Mochales‐Riaño et al. 2023; Khan et al. 2021; Pinto et al. 2024; Saremi et al. 2019). This process often begins with population isolation and decline, which in turn accelerates genetic drift and inbreeding. These factors lead to a reduction in neutral genetic diversity and can exacerbate the accumulation of harmful mutations, ultimately diminishing the population's overall genetic health (Pinto et al. 2024). Our RoH results for the Northern Maned Sloths revealed a trend of rapidly increasing inbreeding levels over the past few generations. Although there are limitations to accurately dating the onset of these increased inbreeding events, it is most likely associated with recent deforestation happening in the northern Atlantic Forest. Thus, our findings highlight how genomics can serve as a powerful tool for monitoring the genomic impacts of deforestation. As a historically large population, the northern lineage may face even greater challenges when subjected to bottlenecks due to the accumulation of masked load. This demonstrates that species that were previously thriving can suffer even greater impacts when threatened. Consequently, genetic load, analysed within an evolutionary framework, should be recognised as a key factor in predicting species viability. As suggested by Dussex et al. (2023), future research utilising multi‐species comparisons and combining a variety of empirical data, ideally fitness data, could explore whether genetic load can serve as a proxy for predicting the likelihood of future reductions in fitness.

While the northern lineage exhibits recent signs of genomic erosion, the southern lineage faces challenges associated with its long‐term small population size. This has resulted in pronounced genomic erosion, including low genetic diversity and higher historical inbreeding levels. This is critical for sloths and other species with limited mobility, as they are particularly vulnerable to the localised effects of inbreeding (Sachdeva et al. 2022), which can accelerate the transition of masked deleterious mutations into a realised genetic load (Pinto et al. 2024). On the other hand, subdivision into small subpopulations can also facilitate purging of deleterious mutations, making selection more effective than with panmixia (Sachdeva et al. 2022). Our findings underscore the critical role of purging deleterious variants in the persistence of small, isolated populations within the southern lineage. Supporting this, a study of 41 mammalian species by van der Valk et al. (2019) demonstrated that species with historically small population sizes and low genetic diversity tend to exhibit lower genetic loads compared to species with large population sizes. They also identified a weak inverse relationship between genetic load and inbreeding, with some species (e.g., cheetah, snow leopard, tiger, wolves) showing low genetic load but high proportions of their genome in runs of homozygosity. Similarly, Robinson et al. (2018) reported that island foxes, which have historically small populations, carry a reduced burden of strongly deleterious recessive alleles compared to individuals from a mainland large outbred population, reducing their risk of inbreeding depression.

Thus, our study showcases how genomic metrics—such as genetic diversity, inbreeding, and genetic load—can inform unique conservation needs of each population. In particular, such analysis was enabled by the application of high‐quality and genome‐wide data, including a chromosome‐level genome (Formenti et al. 2022; Theissinger et al. 2023). It contributes to fulfilling the promise of genomics as a tool to assess extinction risk of species (van Oosterhout et al. 2022), while also highlighting the complex interplay between genetic proxies. We emphasise that these metrics must be interpreted for each species within an evolutionary framework that considers both ancient and recent environmental conditions.

In conclusion, the current genetic diversity distribution of Maned Three‐Toed Sloths is intricately linked with the history of the Atlantic Forest. Historical environmental changes from the Pleistocene era have driven divergence, genetic diversity, and demographic trajectories in populations of both Northern and Southern Maned Sloths. Currently, deforestation poses significant challenges for the species, but reforestation efforts positively impact population recovery and have the potential to reverse negative effects. To mitigate further habitat loss and preserve the genetic integrity of Maned Three‐Toed Sloth populations, it is imperative to effectively enforce protective measures and expand conservation units throughout the Atlantic Forest.

## Author Contributions

L.S.A. and C.J.M. conceived the work and designed the research. L.S.A. performed the bioinformatic analysis. D.D.P. developed the preprocessing pipeline. F.R.M. and F.R.S. provided the samples and revised the genetic results and manuscript. M.H. revised the manuscript. L.S.A. drafted the manuscript with input from all authors. All authors read and approved the manuscript.

## Conflicts of Interest

The authors declare no conflicts of interest.

## Supporting information


**Data S1:** mec70148‐sup‐0001‐Supinfo.pdf.

## Data Availability

The raw sequencing data will be available in Genbank with the NCBI BioProject accession number PRJNA1137335. Script will be available at GitHub https://github.com/Larissa‐Arantes/ManedSlothsPopGen.git. Benefits from this research come from sharing data and results on public databases. All collaborators, including sample providers and stakeholders, are co‐authors.
