# Supplementary material for: Genomic Signatures in Maned Three‐Toed Sloths From Ancient to Recent Environmental Changes in Brazil's Threatened Atlantic Forest
Source: Mol Ecol. 2025 Oct 18;34(23):e70148. doi: 10.1111/mec.70148 (PMC12684302; doi:10.1111/mec.70148)
Supplement: Supplementary file 1 — Data S1: mec70148‐sup‐0001‐Supinfo.pdf. [file MEC-34-e70148-s001.pdf]

MOLECULAR ECOLOGY

Supplemental Information for:

Genomic signatures in Maned Three-Toed Sloths from ancient to recent environmental changes in Brazil's threatened Atlantic Forest

Larissa S. Arantes, Diego De Panis, Flávia R. Miranda, Fabrício R. Santos, Michael Hiller, Camila J. Mazzoni

Table of Contents:

**Figure S1**	Page 2
**Figure S2**	Page 3
**Figure S3**	Page 4
**Figure S4**	Page 5
**Figure S5**	Page 6
**Table S1**	Page 7
**Table S2**	Page 8

MOLECULAR ECOLOGY

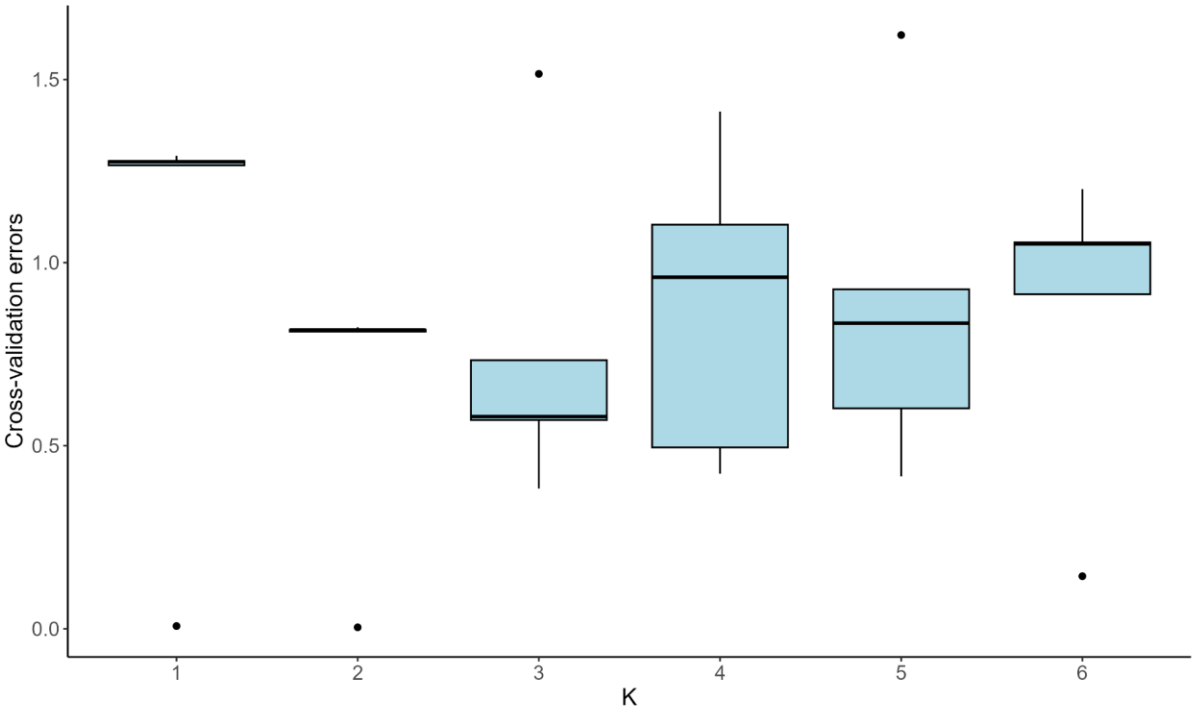

Figure S1 - Cross-validation (CV) error estimation of Admixture analysis for each K. The standard deviation error bars refers to 10 replicates per K. The CV values drop until K = 3.

MOLECULAR ECOLOGY

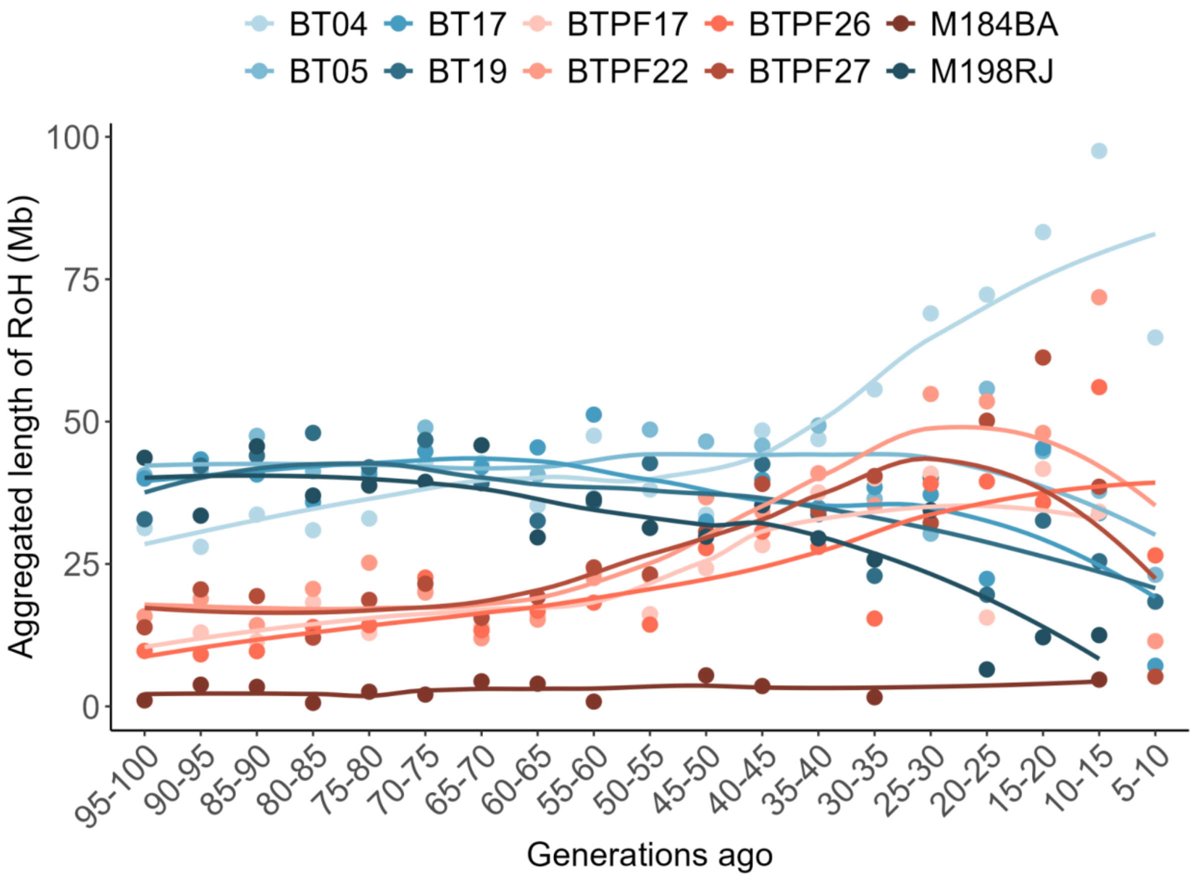

Figure S2 – Distribution of lengths of Runs of Homozygosity (RoH - in Mb) in intervals of five generations and its associated expected number of generations since the individual's maternal and paternal lineages shared a common ancestor for different genome sections. This result was obtained with PLINK2. The line represents a regression (LOESS) fit, capturing the underlying trend in the data. Note that the generation time intervals differ between PLINK2 and Darwindow, as shown in Figure 2B of the main text, due to differences in the sliding window approaches employed by the two tools.

MOLECULAR ECOLOGY

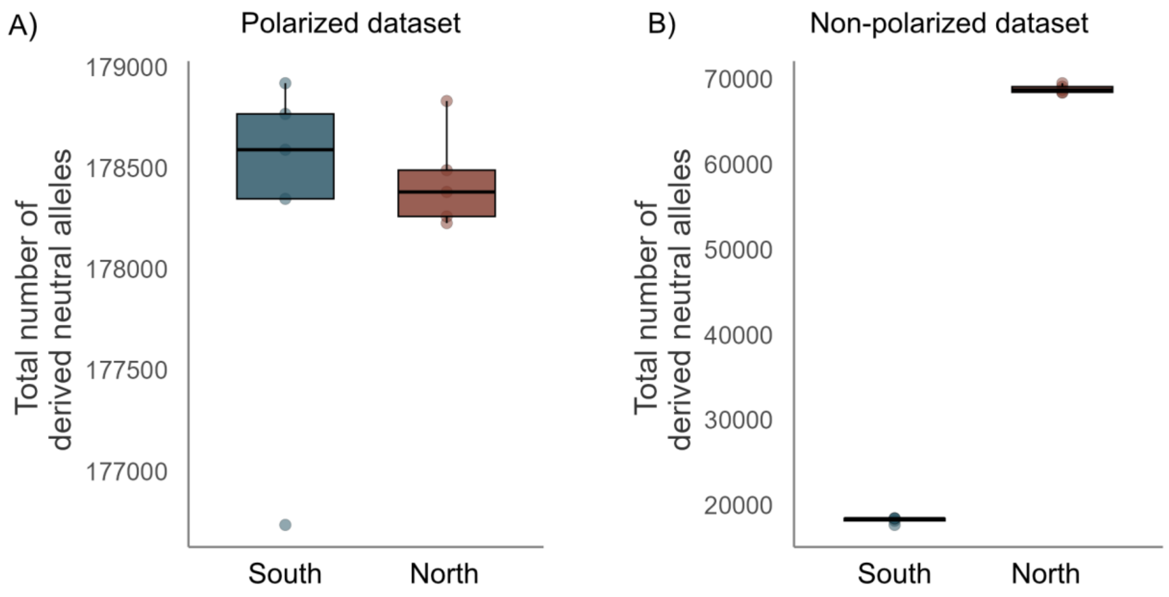

Figure S3 – Number of derived neutral alleles for North and South Maned Sloths, which was calculated for a subset of 100,000 intergenic positions, for the polarized (A) and non-polarized datasets (B). The polarized dataset was generated by redefining the ancestral state of each site in the VCF file based on the consensus allele determined from the outgroup species: Hoffmann's two-toed sloth (*Choloepus hoffmanni*), nine-banded armadillo (*Dasypus novemcinctus*), and Southern tamandua (*Tamandua tetradactyla*).

MOLECULAR ECOLOGY

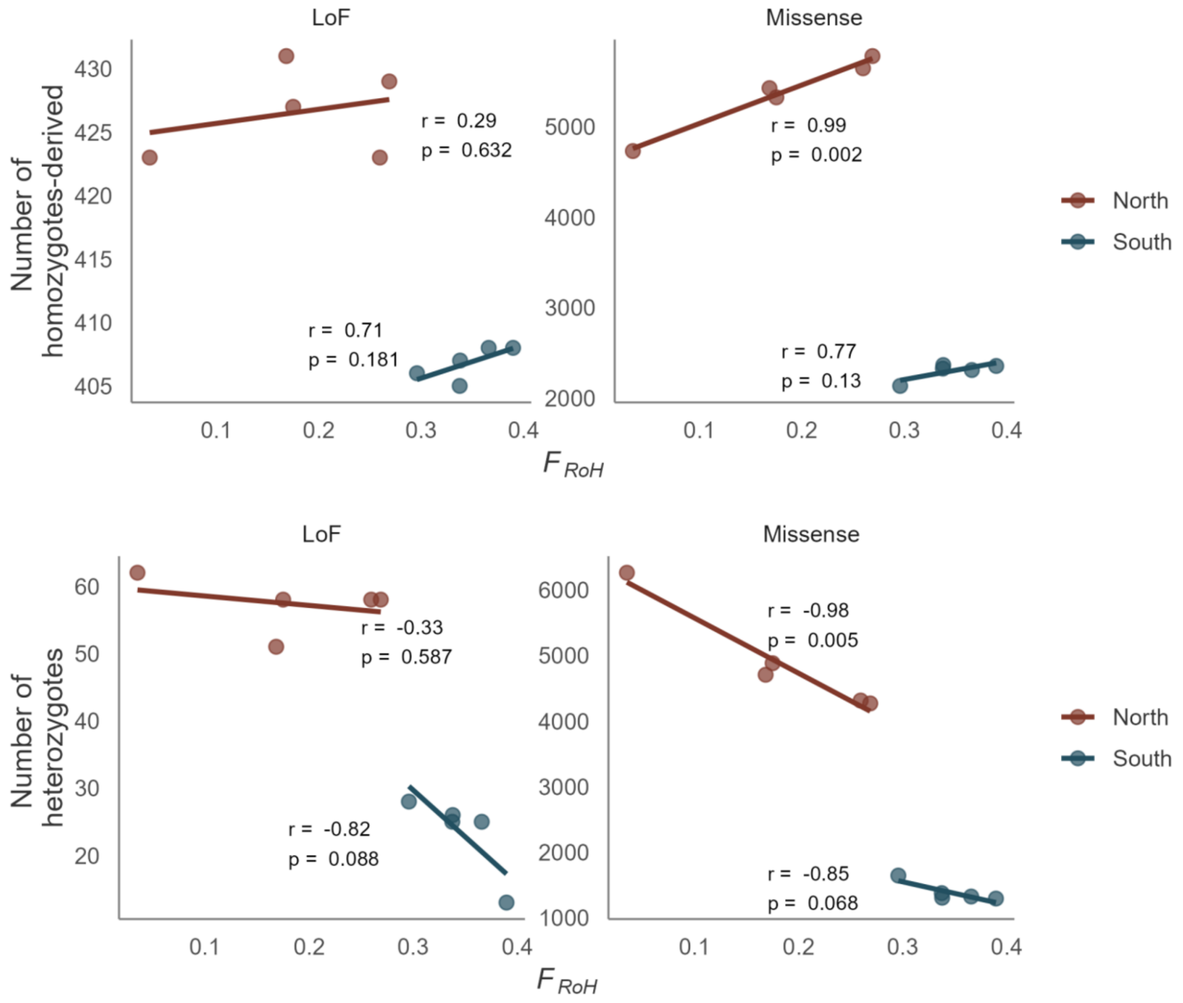

Figure S4 – Relation between inbreeding (F_{RoH}) and number of homozygous-derived and heterozygous genotypes for missense and LoF mutations. Linear regression lines are shown for each lineage. Pearson correlation coefficients (r) and corresponding p-values are provided for each lineage and mutation category tested.

MOLECULAR ECOLOGY

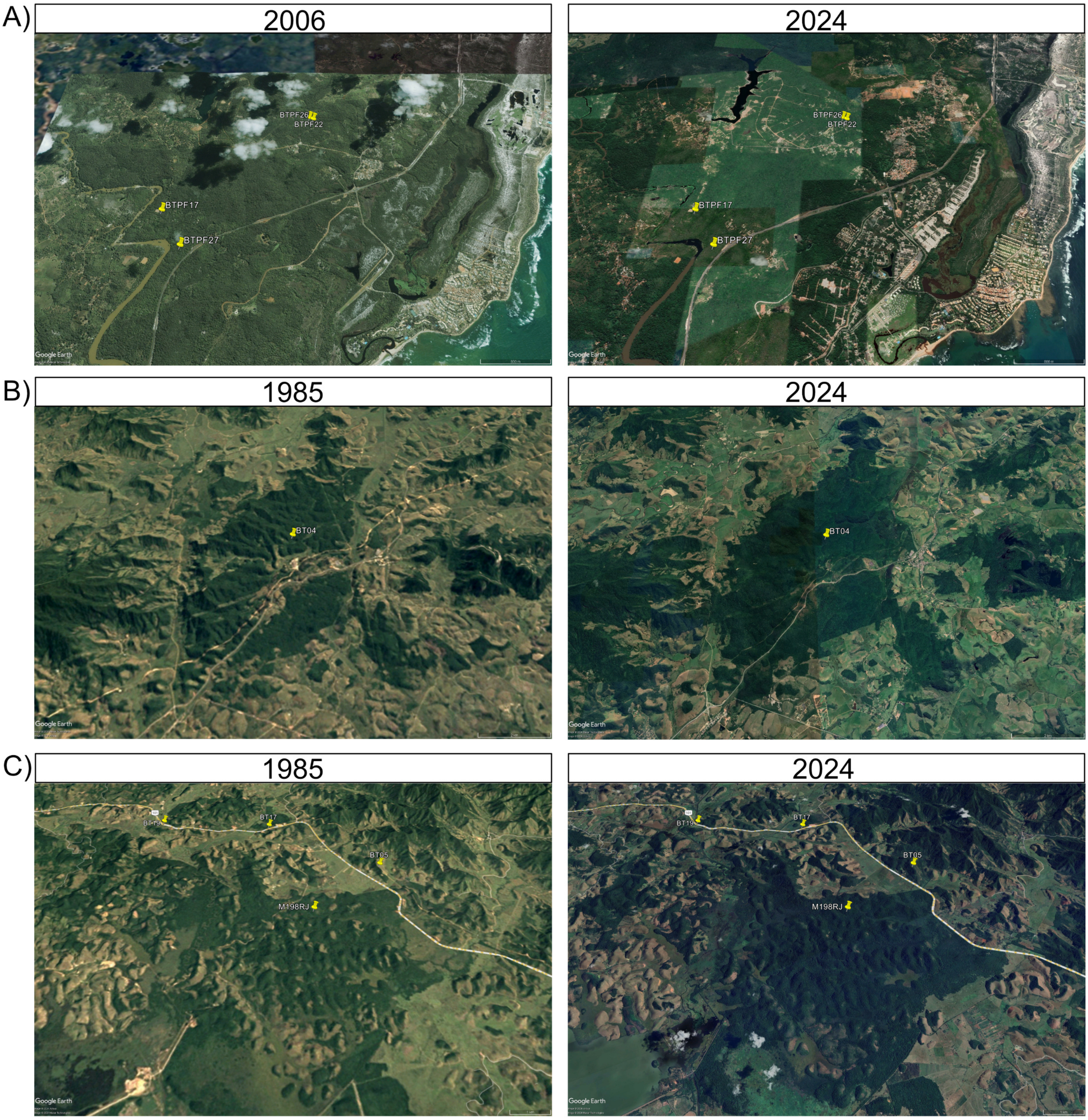

Figure S5 – Comparison between forest coverage in different time points in Atlantic Forest fragments: A) Mata de São João, B) União Biological Reserve, and C) Poço das Antas Biological Reserve, showing the highway BR-101.

MOLECULAR ECOLOGY

Table S1 – Metadata for samples and their respective genomic data used in this study.

Sample ID	Species	Date of Collection	Sex	Age	Location	Detailed location	Geographical coordinates	Coverage	Number of SNPs	SRA accession number
BTPF17	*Bradypus torquatus*	14/09/2020	Unknown	Sub-adult	Mata de São João, Bahia	Ecological Reserve Sapiranga	-12.5669577653177 / -38.0427953933211	18.76	8,909,034	SRR29886158
BTPF22	*Bradypus torquatus*	06/10/2020	Unknown	Sub-adult	Mata de São João, Bahia	Aruá Forest	-12.5510926328098 / -38.0236450784806	20.62	8,878,163	SRR29886157
BTPF26	*Bradypus torquatus*	25/10/2020	Female	Sub-adult	Mata de São João, Bahia	Aruá Forest	-12.5511900148099 / -38.0230740575225	20.11	8,847,413	SRR29886156
BTPF27	*Bradypus torquatus*	28/10/2020	Male	Sub-adult	Mata de São João, Bahia	Ecological Reserve Sapiranga	-12.5721827905504 / -38.0399409474354	27.06	8,918,534	SRR29886155
M184BA	*Bradypus torquatus*	01/08/2003	Male	Unknown	Una, Bahia	-	-15.2 / -39.083333	22.76	9,452,704	SRR29886154
BT04	*Bradypus crinitus*	10/07/2019	Female	Adult	Rio das Ostras, Rio de Janeiro	Biological Reserve União	-22.4181580333522 / -42.0370746360437	22.38	3,311,412	SRR29886163
BT05	*Bradypus crinitus*	09/06/2019	Male	Sub-adult	Silva Jardim, Rio de Janeiro	Igarapé Farm	-22.506435180532 / -42.3083558381588	17.03	3,313,938	SRR29886162
BT17	*Bradypus crinitus*	16/11/2021	Female	Adult	Silva Jardim, Rio de Janeiro	Dois Irmãos Farm	-22.531658551057 / -42.3437813585948	21.65	3,419,957	SRR29886161
BT19	*Bradypus crinitus*	30/11/2021	Male	Adult	Silva Jardim, Rio de Janeiro	BR 101	-22.5641671399466 / -42.3614411171893	23.10	3,415,973	SRR29886160
M198RJ	*Bradypus crinitus*	29/10/2003	Male	Unknown	Silva Jardim, Rio de Janeiro	-	-22.533333 /-42.3	21.29	3,329,542	SRR29886159

MOLECULAR ECOLOGY

Table S2 - Filters applied during the variant calling following GATK's recommended parameter thresholds.

**Filter**	**Threshold**
Sample's Depth (DP)	MIN_DEPTH = 8x and MAX_DEPTH = 2x the individual average depth
Quality by Depth (QD)	< 2.0
Fisher Strand bias (FS)	> 60.0
Mapping Quality (MQ)	< 40
MQRankSum	< -12.5
ReadPosRankSum	< -8.0
ReadPosRankSum	> 8.0
Symmetric Odds Ratio (SOR)	> 3.0
Number of alternate alleles	> 1
Allelic Depth (AD)	0/1 < 20%; 0/1 > 80%; 0/0 or 1/1 >10%
InDels	all

.
